# Effect of ZnO nanoparticles on biofilm formation and gene expression of the toxin-antitoxin system in clinical isolates of *Pseudomonas aeruginosa*

**DOI:** 10.1186/s12941-023-00639-2

**Published:** 2023-10-05

**Authors:** Hassan Valadbeigi, Nourkhoda Sadeghifard, Vahab Hassan Kaviar, Mohammad Hossein Haddadi, Sobhan Ghafourian, Abbas Maleki

**Affiliations:** 1 Clinical Microbiology Research Center, Ilam University of Medical Sciences, Ilam, Iran; 2 Department of Microbiology, School of Medicine, Ilam University of Medical Sciences, Ilam, Iran

**Keywords:** Nanoparticles, Biofilms, Toxin-antitoxin systems, *Pseudomonas aeruginosa*

## Abstract

**Background:**

Biofilm formation by *Pseudomonas aeruginosa* (*P. aeruginosa*) is known to be characteristic of this organism. This bacterium is considered one of the most life-threatening bacteria and has been identified as a priority pathogen for research by WHO. Biofilm-producing *P. aeruginosa* is a concern in many parts of the world due to antibiotic resistance. Alginate also plays an important role in the biofilm formation of *P. aeruginosa* as well as the emergence of antibiotic resistance in biofilms. In addition, the systems of toxin-antitoxin( TA) play an important role in biofilm formation. Metal nanoparticle(NP) such as zinc oxide (ZnO) also have extensive biological properties, especially anti-biofilm properties. Therefore, this study was conducted in relation to the importance of zinc oxide nanoparticles (ZnO NPs) in biofilm formation and also the correlation of gene expression of TA systems in clinical isolates of *P. aeruginosa.*

**Methods:**

A total of 52 *P. aeruginosa* isolates were collected from burns (n = 15), UTI (n = 31), and trachea (n = 6) in hospitals in Ilam between May 2020 and October 2020. Biofilm formation was assessed using a microtiter plate assay. MIC and sub-MIC concentrations of ZnO NPs (10–30 nm with purity greater than 99.8%) in *P. aeruginosa* were determined. Subsequently, biofilm formation was investigated using sub-MIC concentrations of ZnO NPs. Finally, total RNA was extracted and *RT- qPCR* was used to determine the expression levels of genes of *mazEF*, *mqsRA*, and *higBA* of TA systems.

**Results:**

Six isolates of *P. aeruginosa* were found to form strong biofilms. The results showed that ZnO NPs were able to inhibit biofilm formation. In our experiments, we found that the sub-MIC concentration of ZnO NPs increased the gene expression of antitoxins *mazE* and *mqsA* and toxin *higB* of TA systems treated with ZnO NPs.

**Conclusions:**

In the present study, ZnO NPs were shown to effectively inhibit biofilm formation in *P. aeruginosa.* Our results support the relationship between TA systems and ZnO NPs in biofilm formation in *P. aeruginosa*. Importantly, the expression of antitoxins *mazE* and *mqsA* was high after treatment with ZnO NPs, but not that of antitoxin *higA*.

## Background

One of the concerns with opportunistic pathogens in human is antibiotic resistance in biofilm-producing *Pseudomonas aeruginosa* [1, 2]. So, antibiotic-resistant biofilms are the major cause of *P. aeruginosa*-associated infections and lead to increased morbidity and mortality. Biofilms are a surface-associated bacterial community that plays an important role in chronic infections, such as cystic fibrosis, burn wounds, bacterial keratitis, urinary tract infections, and peritoneal dialysis catheter infections, as well as acute infections [3].

Bacteria such as *P. aeruginosa* are protected by biofilms from various environmental stresses such as antimicrobial agents and antibiotics [4] So, due to increasing resistance to antimicrobial agents, *P. aeruginosa* still remains an infectious disease when it forms biofilms or is absorbed into a host. Biofilms are also able to adhere to surfaces, making them harder to remove. This means that *P. aeruginosa* can persist in the environment and be a source of recurrent infections. Therefore, it is important to take preventive measures to contain the spread of this pathogen.

hence, it is important to develop strategies to prevent and control of antibiotic resistance in biofilm-producing *Pseudomonas aeruginosa*.

The use of nanotechnology to produce new nanomaterials for use in medicine has opened up a new world of possibilities [5].A metal nanoparticles has a variety of properties that make it suitable for medical applications, which is why it is considered an effective antibacterial agent. Therefore, ZnO NPs have been extensively studied due to their extensive biological activity. Zinc oxide is the most commonly used zinc nanoparticle [6] because it has less toxic properties and is more effective against resistant microbial pathogens. They also have selective toxicity against bacteria [7]. There are several noteworthy properties of this nanoparticle, including its chemical and physical stability, high catalytic activity, and effective antibacterial activity. Moreover, some metal nanoparticles of ZnO NPs were reported to possess anti-biofilm properties [8, 9].

The antibacterial and biofilm inhibitory properties of ZnO NP have been widely reported against a variety of microbes, including *P. aeroginosa*, *Streptococcus pneumoniae*, *Listeria monocytogens*, *Salmonella enteritidis*, and *E. coli* [10].

Due to their potent antimicrobial activity, these NPs can reduce microbial adhesion, proliferation, and biofilm growth. ZnO NPs damage bacterial cells through the formation of reactive oxygen species (ROS) [11].

Alginate is a polysaccharide that plays an important role in biofilm formation in *P. aeruginosa*. Alginate is also involved in antibiotic resistance of biofilms and helps to protect bacteria from antibiotics [12]. Structurally, alginate forms a polymer containing α-L-guluronic acid and β-D-mannuronic acid, which is encoded by *algD*. *algD* is located in a large operon and is necessary for alginate production, and its expression is fully controlled [12, 13]. It means that AlgD is the key enzyme that catalyzes the formation of alginate polymers. Thus, AlgD plays a central role in the formation of alginate polymers, making it a key enzyme for alginate production.

According to a number of studies, *P. aeruginosa* has TA systems that regulate biofilm-associated genes such as *MqsR/MqsA* [14]. In addition, hundreds of genes are differentially regulated during biofilm development, including quorum sensing (*lasIR*, *rhlIR*), *psl*, and *pel*.

The bacterial TA systems have many physiological functions such as apoptosis, growth arrest, gene regulation, and survival. These systems have retained the genetic element as an addiction module [15].TA systems consist of two genes in an operon encoding a stable toxin moiety and a labile antitoxin moiety encoded on either extrachromosomal units or chromosomal units. A chromosomally encoded TA system is critical for cell viability and plasmid stability, whereas an extrachromosomally encoded TA system is important for biofilm formation, persister cell formation, growth arrest, and tolerance to multiple drugs. The chromosomal and extrachromosomal TA systems consist of the same two components, but in different configurations. Both modules are necessary for the proper functioning of the cell [16]. Moreover, TA systems are classified into different types according to the nature and mode of action of the antitoxin; the type II TA systems is the most common compared with the other types [16]. The type II TA system is responsible for most cases of antibiotic resistance, making it an important target for research and development of new antibiotics.

It is also an important tool to gain insight into the functioning of other TA systems. The *MqsR*/*MqsA* pair was the first TA system associated with biofilm formation and regulated biofilm formation [17, 18]. The role of genes of the TA system in biofilm formation, such as *MazEF*, *RelBE*, *higBA*, has also been investigated [18],On the other hand, antibiotics targeting *P. aeruginosa* are not effective against its ability to form a biofilm. Therefore, the present study investigated the effect of ZnO NPs as antimicrobial agents on *P. aeruginosa* biofilm formation and also the correlation of gene expression of TA systems among clinical isolates.

## Methods

### Bacterial strains, chemicals, and growth conditions

A total of 52 *P. aeruginosa* isolates were collected from Ilam hospitals between May 2020 and October 2020. The isolates were from patients with burn infections (n = 15), urinary tract infections (UTI) (n = 31), and tracheal infections (n = 6). The collected samples were identified by routine biochemical and microbiological tests such as catalase, oxidase, citrate, MR-VP, indole and OF. In addition, *P. aeruginosa* PAO1 was used as the standard strain in this study.

### Preparation of ZnO NPs and detection of MIC and sub-MIC concentration of ZnO NPs in ***P. aeruginosa***

#### Preparation of ZnO nanoparticle suspension

ZnO NPs were purchased in Kara Pajuhesh Amirkabir. ZnO NPs have a size of 10–30 nm and purity of over 99.8%. To prepare ZnO nanoparticle suspensions, 32 milligrams of ZnO NPs were added to 1000 ml of sterile water and shaken vigorously. The suspension solution was treated with ultrasound (100 W, 40 kHz). ZnO nanoparticle powder was mixed with dimethyl sulfoxide (DMSO) as solvent to prevent clumping of nanoparticles [19]. concentrations below 8000 μg /ml ZnO.

#### Detection of MIC and sub-MIC

The serial dilution method (ranged from 128 to 16,000 μg/ml) was used for MIC of ZnO NPs (volume 150 μl per well ). In the first step, 50 μl Mueller-Hinton Broth (MHB) medium was added to a 96 well microplate. Then, different concentrations (16, 32, 64, 128, 256 gr/1000μl) of ZnO NPs were added. Finally, 50 μl a bacterial suspension with 0.5 McFarland standard turbidity was added to the wells and incubated at 37 °C for 24 h. Subsequently, the MIC and sub-MIC were determined according to the lowest concentration at which growth was inhibited by the ZnO NPs [20]. *P. aeruginosa* PAO1 was used as a positive control.

### Detection of biofilm production and anti-biofilm activity of ZnO nanoparticles

The George A. O’Toole protocol was evaluated for biofilm formation ability using a microtiter plate assay. The microtiter plate assay was performed in triplicate and the average of the three wells was calculated for each strain. Strains were classified as follows: A ≤ Ac = no biofilm producer, Ac < A ≤ (2 × Ac) = weak biofilm producer, (2 × Ac) < A ≤ (4 × Ac) = moderate biofilm producer and (4 × Ac) < A = strong biofilm producer [27].

Six strong biofilm-producing *P. aeruginosa* isolates and one PAO1 strain were subjected to biofilm study using ZnO nanoparticles.

### Total RNA extraction

RNA was extracted using a total RNA extraction kit (DENAzist, IRAN) according to the manufacturer’s instructions. Briefly, a single colony from each bacterium was selected and inoculated into 5 ml of LB broth, followed by incubation at 37 °C overnight. One hundred microliters of the liquid cultures were inoculated into 10 ml of fresh LB broth and grown to mid-exponential phase (optical density at 600 nm of approximately 0.5) at 37 °C with shaking at 185 rpm. Finally, RNA was extracted using a total RNA extraction kit.

In the next step, cDNA was synthesized using the Easy TM cDNA Synthesis Kit (Parstous) according to the manufacturer’s instructions. After determination of the cDNA concentration using nonodrop (TITERTEK, BERTHOLD, Germany), the final cDNA concentration was the same for all.

### Expression genes of toxin and antitoxin systems by quantitative real-time PCR (RT -qPCR)

The Gen Script-Real Time PCR Primer Design Tool was used to design primers forward and reverse for *algD* and for the *mazEF*, *mqsRA*, and *higBA* TA system and *gyrB* genes. The *gyrB* gene from *P. aeruginosa* was used as a reference gene. RT- qPCR was performed using the Syber Green PCR Master Mix [21]. Gene expression levels of *algD*, *mazEF*, *mqsRA*, and *higBA* were measured as the gene expression ratio between the target gene and the reference gene by relative quantification. Primers used for PCR amplification are listed in Table 1.

**Table 1:** Oligonucleotide primers used for RT- qPCR in this study. *mazEF*, *mqsRA*, and *higAB* (TA system), *algD* (alginate), *gyrB* (housekeeping) in *P. aeruginosa*

TA system	${\rm{Primers}}\,\left( {{\rm{5'}}\, \to \,{\rm{3'}}} \right)$	Amplicon Size
*mazE*	F: GACGATAGCCAGCTTTGTCG R: CCGCCTCGTTGATTTCTTCG	77
*mazF*	F: CGCCCTAGCCATTCCTATCA R: TGCAGGGCTTCATCTATGGT	192
*mqsR*	F: ACTGCATGGACGAAGAGGAT R: TCAGCTTCAAACCAGGGACT	112
*mqsA*	F: CGCACAAAGCAAACACAACA R: TCTAGTGGCTCCGCTTTCAT	181
*higA*	F: AGGGTACGAATCACGGCTAT R: AGATCATCTGCTGCTCAACG	150
*higB*	F: AATATCCCTTAGCAATCGTGTTC R: TGAAAGATGCTGCGGAAA	78
*algD*	F: GGCTATGTCGGTGCAGTATG R : GGCTATGTCGGTGCAGTATG	72
*gyrB*	F: CTGCTTCACCAACAACATCC R: GGTGGCGATCTTGAACTTCT	130

### Statistical analysis

Statistical analysis was performed using SPSS for Windows software (Version 18 software package SPPSS Inc.). The mean of the results was analyzed by analysis of variance (one-way ANOVA) with a probability level of less than 0.05.

## Results

### Detection of biofilm production

A total of 52 *P. aeruginosa* were isolated, 6 isolates were selected because of their strong biofilm production. The isolates that were capable of moderate and weak biofilm producers were deleted in this study (Table 2).

**Table 2:** Biofilm producing *P. aeruginosa*

	Biofilm			
Sample	Weak	Moderate	Strong	Total
UTI	19.4%(n = 6)	51.6%(n = 16)	29.0%(n = 9)	31
Burn	26.6%(n = 4)	53.3%(n = 8)	20%(n = 3)	15
Tracheal	16.66%(n = 1)	50%(n = 3)	33.33%(n = 2)	6

### MIC and sub-MIC of ZnO NPs by serial dilution method

Six isolates, including UTI = 2, Burn = 2, and Tracheal = 2, which formed strong biofilms, were selected for study. The results of the serial dilution method showed that the MIC of ZnO NPs was 8000 μg/ml in six isolates that formed biofilms. Also, concentrations of 4000 μg/ml(sub-MIC), were used for evaluation anti-biofilm activity of ZnO NPs.

### Anti-biofilm activity of ZnO NPs against biofilm producing ***P. aeruginosa***

The anti-biofilm activities of ZnO NPs were evaluated against six isolates, such that they were able to inhibit biofilm bacterial growth at concentrations below 8000 μg /ml ZnO NPs. Consequently, all isolates were inhibited by ZnO NPs in OD (0. 22). *P. aeruginosa* PAO1 was used as a positive control with OD (2.54).

### The effect of sub-MIC of ZnO NPs on the expression levels of toxin and antitoxin system genes

**Fig. 1:**
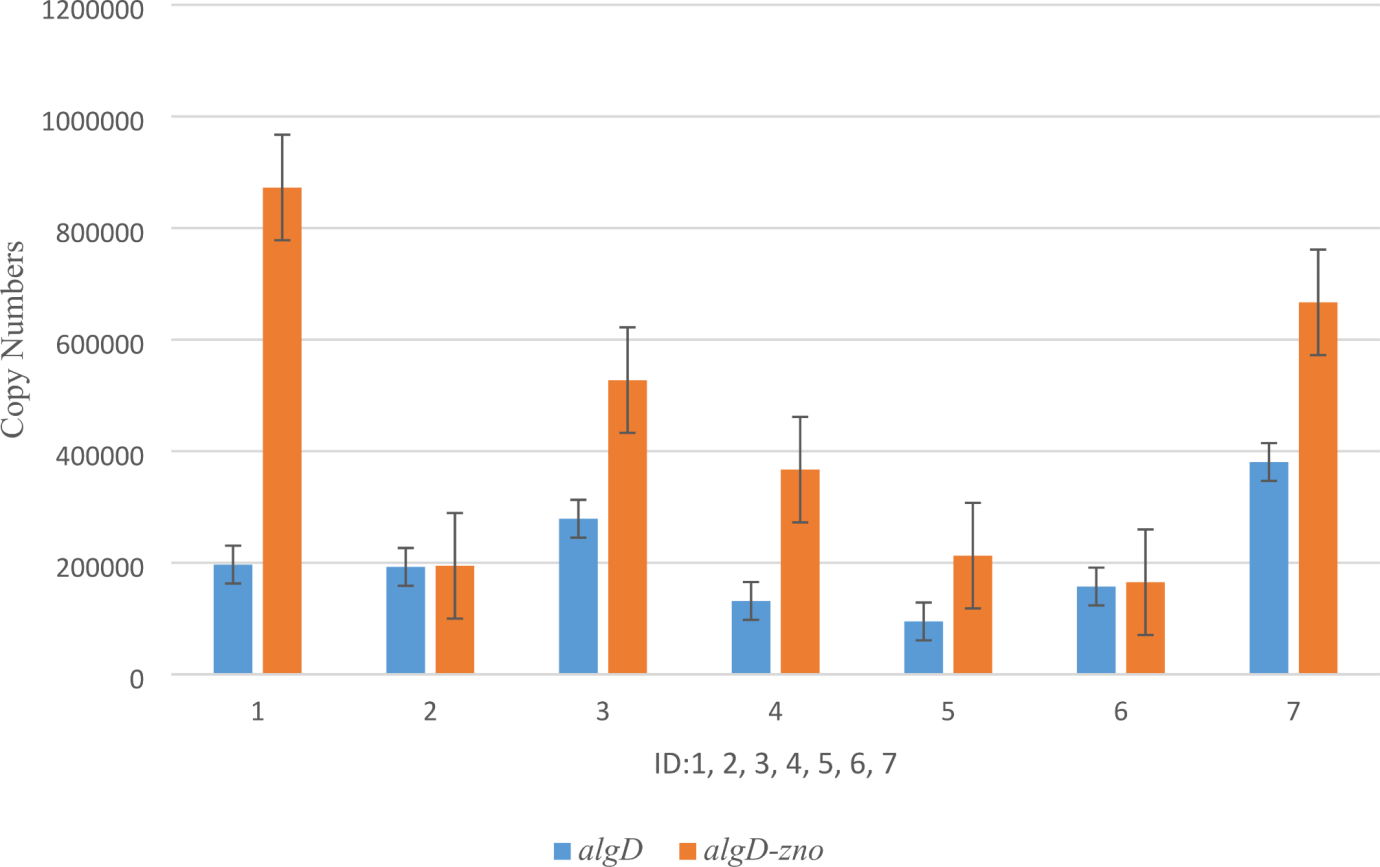
The effect sub-MIC of ZnO NP_S_ on expression level of *algD* gene. ID: Urine:1,2, Burn: 3,4, Trachal:5,6 and PAO1:7

The isolates of biofilm-producing *P. aeruginosa* inhibited by ZnO NPs at sub-MIC concentrations were selected for the current study. Real-time qPCR results showed that the gene expression of *algD* and also the genes of TA systems such as *mazEF*, *mqsRA* and *higBA* were different in these isolates. It was found that the expression of *algD* gene increased when exposed to sub-MIC concentrations of ZnO NPs in comparison to *algD* gene alone(Fig. 1.), although this increase was not statistically significant (P-value < 0.05).

It was found that the expression of *algD* gene increased more than that of *algD* gene when exposed to sub-MIC concentrations of ZnO NPs.

**Fig. 2:**
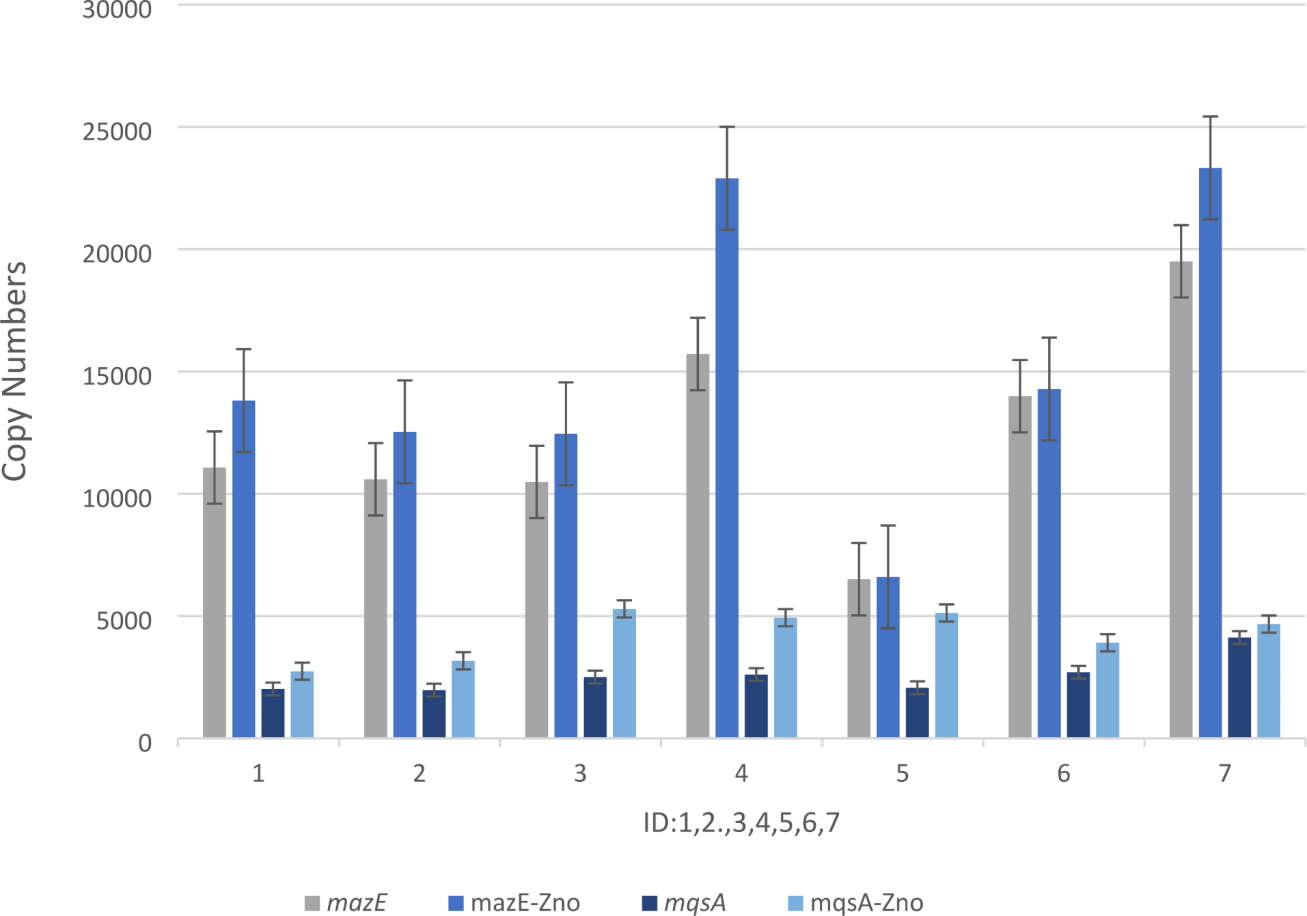
The effect sub-MIC of ZnO NP_S_ on expression level of the Antitoxin *mazE* and *mqsA* of TA- system. ID: Urine:1,2, Burn: 3,4, Trachal:5,6 and PAO1:7

**Fig. 3:**
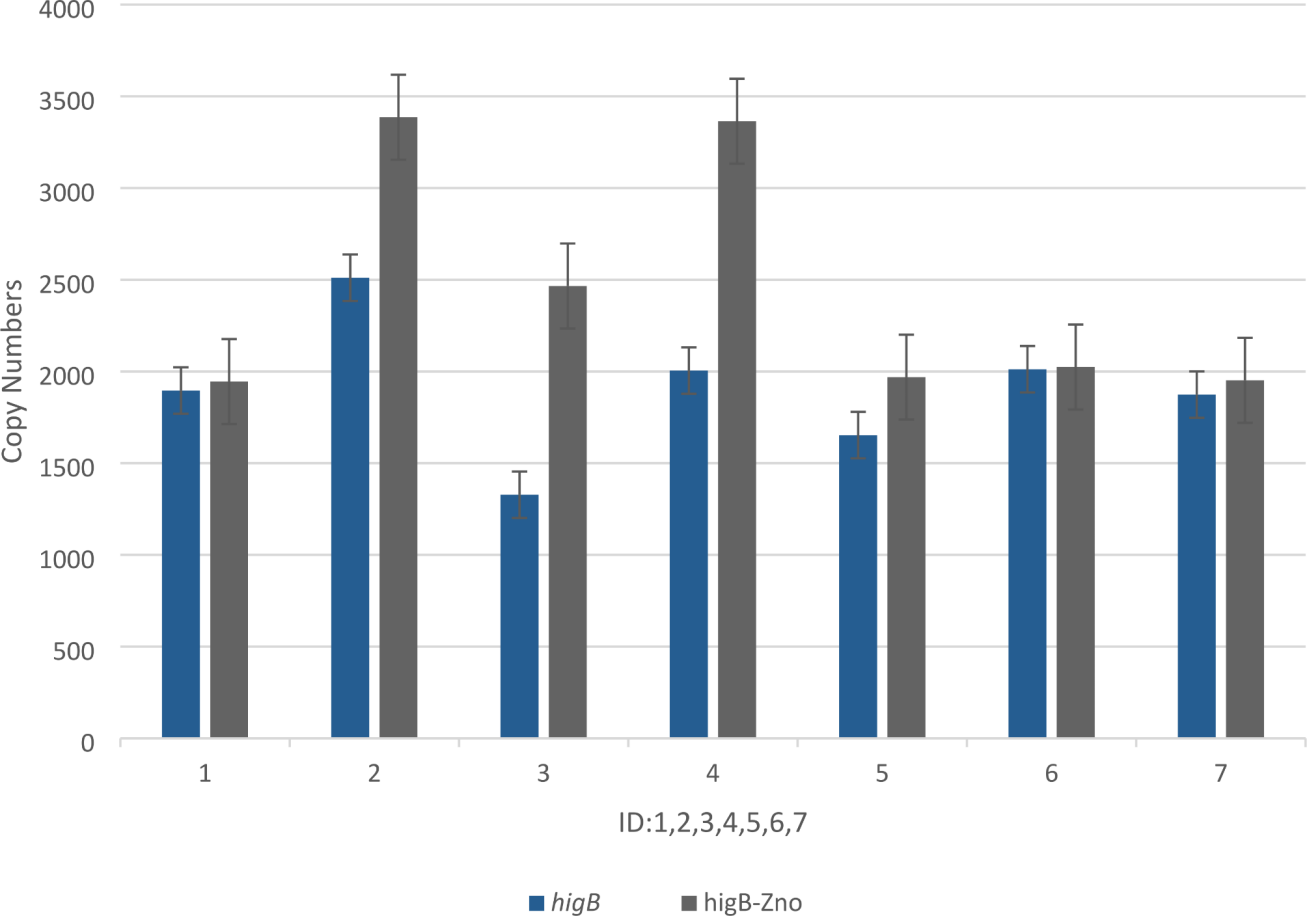
The effect sub-MIC of ZnO NP_S_ on expression level of the toxin *higB* of TA- system. ID: Urine:1,2, Burn: 3,4, Trachal:5,6 and PAO1:7

As a result, the antitoxin *mazE* was highly expressed in biofilms formed by *P. aeruginosa* at sub-MIC concentrations of ZnO NPs(Fig. 2.), whereas the toxin *mazF* was barely expressed. The expression of antitoxin *mqsA* treated with sub-MIC concentrations of ZnO NPs was also higher compared with antitoxin *mqsA*(Fig. 2.). However, the expression of antitoxin *higA* and *higA* treated with sub-MIC concentrations of ZnO NPs was negligible; instead, the expression of toxin *higB*(Fig. 3.), treated with sub-MIC concentrations of ZnO NPs was significant compared with *higB* (P-value < 0.043) (Table 3).

**Table 3:** Mean, standard deviation, and P-value of the TA system genes and the *algD* gene upon treatment with sub-MIC concentrations of ZnO NP_S_ in biofilm-producing *P. aeruginosa* isolates from burns, urine, and trachea

Genes of toxin antitoxin systemes	Isolates	Numbers	Mean.	Std. Deviation	Sig.
*mazE* & *mazE*-ZnO	Urine	1 to 31	2290.286	2360.112	0.042
	Burn	32 to 47			
	Trachea	48 to 54			
*higB &higB-ZnO*	Burn	1 to 31	546.714	566.239	0.043
	Trachea	32 to 47			
	Urine	48 to 54			
*mqsA* &*mqsA-ZnO*	Burn	1 to 31	1692.429	1014.040	0.004
	Trachea	32 to 47			
	Urine	48 to 54			
**Genes**	**Isolates**	**Isolates**	**Mean.**	**Std.** **Deviation**	**Sig.**
*algD* & *algD-ZnO*	Burn	1 to 31	186513.857	243814.989	0.05
	Trachea	32 to 47			
	Urine	48 to 54			

## Discussion

Biofilm formation is the hallmark of *P. aeruginosa*, an important opportunistic bacterial pathogen that can colonize surfaces [1]. Clinically, biofilms play an important role in persistent and chronic infections by reducing the immune response and antibacterial efficacy [22]. The search for new agents that target biofilms could lead to new strategies for controlling infections and provide solutions to biofilm-related problems. On the other hand, the rate of biofilm formation by *P. aeruginosa* isolates from patients at different sites in Iran varied from 43.5 to 99.5% (86.5% overall) [12]. Accordingly, in the current study, we investigated the biofilm formation ability of *P. aeruginosa* bacteria isolated from UTI, burn and tracheal specimens from hospitalized patients in Ilam hospitals, and the activity of ZnO NPs on *P. aeruginosa* biofilm formation. Despite the fact that all the tested bacteria were capable of forming biofilms, six isolates were selected as being capable of forming strong biofilms.

Nanoparticles such as copper, silver, nickel, and zinc have been shown to be active against bacteria, so some of them are also effective in eliminating bacterial biofilms [5]. On the other hand, the biofilm interactions between nanoparticles and bacteria are poorly understood and, more importantly, little is known about the molecular mechanisms underlying the antimicrobial effects of nanoparticles. Furthermore, nanomaterials can interact with bacteria and affect bacterial life cycles, which in turn can alter bacterial activities such as cell-cell communication [23]. In recent years, various methods have been developed to eradicate biofilms. Some nanoparticles, such as Zn and ZnO NPs, have bactericidal activity and remarkable therapeutic efficacy in killing biofilm-producing bacteria. Moreover, ZnO NPs are used in products such as paints, cosmetics, sunscreens, and food as a food source [24] Antimicrobial nanoparticles offer many advantages over conventional antibiotics in terms of reducing acute toxicity, overcoming resistance, and reducing cost [25]. In the current study, ZnO NPs were used at sub-MIC concentration. Our studies showed that ZnO NPs at sub-MIC concentrations and above exhibited antibiotic activity against biofilm-producing *P. aeruginosa* and were able to inhibit the growth of biofilm bacteria at these concentrations. Several studies have demonstrated the antibacterial properties of ZnO nanoparticles. The effect of ZnO NPs with a size of 30–90 nm against *P. aeruginosa* and MIC at a concentration of 300 μg/ml was reported by Saadat et al [26]. The results of Lee et al. showed that ZnO NPs can inhibit biofilm formation and virulence factor production in *P. aeruginosa* [7].

A number of bacteria, including *Escherichia coli*, *Pseudomonas chlororaphis*, *Pseudomonas putida*, and *Staphylococcus aureus*, have been tested for their antibacterial and antibiofilm properties by using ZnO NPs [27–32]. However, this study is the first to investigate the inhibitory effect of ZnO NPs on genes related to biofilm formation and TA systems of *P. aeruginosa*.

According to findings of this study, we found that there was a correlation between the degree of biofilm formation and the expression of the gene *algD* and ZnO NPs. This means that ZnO NPs are able to increase the expression of *algD*, which is one of the most important genes in biofilm development.

As we know, there are different types among TA systems, of which type II is the most famous and well-known [14] so, TA systems such as *higBA*, *mazEF*, and *mqsRA* can be potentially associated with biofilm formation [33]. Different studies showed a correlation of TA systems and biofilm formation. According to Gonzalez Barrios et al., *mqsRA* plays an important role in motility and biofilm formation [34]. Kasari et al. [35] also confirmed the association between the *mqsRA* gene and biofilm formation. Our study revealed that, ZnO NPs strongly enhanced the expression of toxin *higB* expression in biofilms of *P. aeruginosa* compared with toxin *higB* alone. Our results showed that ZnO NPs increased the expression of antitoxin *mazE* and *mqsA* compared with antitoxin *mazE* and *mqsA* that were not treated with ZnO NPs. In contrast, the expression of the ZnO nanoparticle-treated toxins *mazF* and *mqsR* and antitoxin *higA* was insignificant. So, expression of genes antitoxin *higA* decrease while toxin *higB* increased so that this issue was interesting because of increasing of toxin. On the other hand, burn isolates expressed significant levels of *mqsA* and *higB* genes. Therefore, this study indicated that ZnO NPs reduced biofilm formation of *P. aeruginosa* especially in the burn isolates and increased the expression of the antitoxins *mazE* and *mqsA* and the toxin *higB* TA system in these bacteria.

## Conclusion

The findings of this study suggest that ZnO NPs inhibit biofilm formation in *P. aeruginosa* and may have potential as anti-biofilm agents. Moreover, our results underscored the effect of ZnO NPs on biofilm formation and their relationship with TA systems in *P. aeruginosa*. The antitoxin *mazE* was strongly expressed upon treatment with ZnO NPs, whereas *mazF* was suppressed, as were the antitoxin *mqsA* and the toxin *mqsR*, especially in burn isolates. Importantly, the antitoxin *higA* was not expressed in response to exposure to ZnO NP compared with *higB*. Thus, ZnO NPs could be useful in controlling the expression of TA systems in *P. aeruginosa* and as anti-biofilm agents. Nevertheless, the behaviour of TA systems in relation to the expression of various genes is confusing. Consequently, further research is needed to determine the potential of ZnO NPs in the expression of other genes and other types of TA systems as well as other biofilm-generated infections in *P. aeruginosa.*

## Data Availability

All data is in article.

## Abbreviations

DMSO: Dimethyl sulfoxide
MHB: Mueller-hinton broth
MIC: Minimum inhibitory concentration
*P. aeruginosa:*: Pseudomonas aeruginosa
RT- qPCR: Quantitative real- time PCR
TA systems: Toxin-antitoxin systems
UTI: Urinary tract infections
WHO: World health organization
ZnO NPS: ZnO nanoparticles
ZnO: Zinc oxide
